# Tobacco and its Relationship with Oral Health

**DOI:** 10.31729/jnma.6605

**Published:** 2021-11-30

**Authors:** Anima Bhandari, Nisha Bhatta

**Affiliations:** 1 Kathmandu University School of Medical Sciences, Dhulikhel, Nepal

**Keywords:** *mouth neoplasms*, *oral health*, *smoking*, *tobacco*

## Abstract

Tobacco and its various forms cause major oral health problems. Tobacco either in smoked or smokeless forms is prevalent in Nepal and counts as a risk factor for the causation of various red and white lesions, premalignant lesions, oral cancers, gingival and periodontal diseases. Tobacco in conjunction with other risk factors adds a potential threat to oral diseases and its timely control is a cure to those threats. This article focuses on tobacco and its forms affecting oral health and also focuses on its prevention and control from the ground to the National level.

## INTRODUCTION

Tobacco consumption is one of the major public health problems in the world. Annually, 27,100 premature deaths are attributed to tobacco-related diseases in Nepal. Tobacco along with its harm to oral and overall systemic health also causes a potential threat to human life as well.^1^ About 24% of the adult population use tobacco as per data suggested by WHO in 2018 killing more than 8 million people per year around the world. About 80% of the total tobacco users are from low- and middle-income countries like Nepal.^2^ Nepal being a developing nation with a low socioeconomic status tobacco use has been a deep-rooted problem. The prevalence of tobacco use is 56.5% in men and 19.5% in women which is higher than in other countries. The figure is higher in marginalized areas of the country.^3^ About 14.9% of total mortality (27,100 deaths annually) are attributed to tobacco use in Nepal.^4^

A total of 32 studies and 5 policy documents were reviewed. Findings suggest that tobacco consumption was higher among men, illiterates, older people, people living in rural and mountainous areas, and those who initiated smoking as adolescents. Peer pressure and parental/family smoking were major contributing factors for tobacco initiation.^4^ Cigarette smoking, bidi, khaini, areca nut, slaked lime, snuff, gutkha, paan, hookah, chillum, kankad, sulfa; the major form being paan with tobacco and is most popular in the Terai region. Low socio-economy, illiteracy, unskilled manpower, socio-cultural support to some forms of tobacco is tobacco use boosters in Nepal. Its prevalence in teenagers and young adults is no less. Peer pressure, imitation, fantasy, advertisements, and stress encourage todays' adults to have such habits. This ultimately causes oral diseases, malignancy, and even deaths and sadly the future appears worse.

## TOBACCO AND ORAL HEALTH

All of the major forms of tobacco used in Nepal have oral health consequences. Both smoked and smokeless tobacco are prevalent in Nepal. Cigarette smoking can cause a spread of adverse oral effects, including gingival recession, impaired healing following periodontal therapy, oral carcinomas, mucosal lesions (e.g., oral leukoplakia, nicotine stomatitis), periodontal disease, premature tooth loss, and tooth staining. The use of smokeless tobacco is associated with increased risks of oral cancer and oral mucosal lesions, Oral cancer being the second most common cancer in Nepal and sixth among the cancer deaths.^5^ Smokeless tobacco use also causes oral conditions like gingival keratosis, tooth discolouration, halitosis, enamel erosion, gingival recession, alveolar bone damage, periodontal disease, coronal or root-surface dental caries due to sugars added to the product, and tooth loss.^4^

When tobacco is smoked, nicotine rapidly reaches peak levels in the bloodstream and enters the brain; if the smoke is not directly inhaled into the lungs, nicotine is absorbed through mucous membranes and reaches peak blood levels and therefore the brain more slowly.

Although cigarettes are the most commonly used form of tobacco, other recreational tobacco formulations include conventional smokeless tobacco; compressed dissolvable tobacco; cigars; tobacco pipes and water pipes (i.e., hookahs); and electronic cigarettes (e-cigarettes).^4^

## TOBACCO CONTROL IN NEPAL

Nepal signed the WHO Framework Convention on Tobacco Control (WHO FCTC) on 3 December 2003 and ratified it on 7 November 2006. The Council of Ministers of the Government of Nepal passed an Executive Order in 1992 and 2010 on tobacco-free initiatives such as the implementation of health warnings on tobacco products; prohibition of publicly smoking, workplaces, and on public transport; collecting health tax from tobacco industries for the treatment of diseases caused by tobacco use; allocation of funds to disseminate information and conduct education and communication activities on the harmful effects of tobacco use, and enforce a ban on tobacco advertisements through hoardings. The government, non-government and private sectors are involved.^3^

The government levies excise on tobacco products per annum and on import and customs for international brands of tobacco products. Nepal has been cooperating with regional and global tobacco control networks in the transfer of technical, scientific, and legal expertise and technology, as mutually agreed, to establish and strengthen national tobacco control strategies, plans, and programs.^3^

## CESSATION COUNSELLING

Due to the oral health implications of tobacco use, dental practices might provide a distinctively effective setting for tobacco use recognition, prevention, and cessation. Overall healthcare professionals, including dental professionals, can help smokers quit by consistently identifying patients who smoke, advising them to quit, and offering them information about cessation treatment. The U.S. Department of Health and Human Services and Agency for Healthcare Research and Quality has published a 5-step algorithm for healthcare professionals to use when engaging patients who are dependent on nicotine called "the 5As".^6^ The 5 steps are as follows:

**Ask:** Problem identification and documentation of tobacco use status for every patient at every visit.

**Advise:** In a strong, clear, and personalized manner, urge every tobacco user to quit.

**Assess:** Is that smoker willing to form a quit attempt this time? If yes, proceed to the next step, if no 5R principle is used.

**Assist:** For the patient willing to make a quit attempt, use counseling and pharmacotherapy to help him or her quit.

**Arrange:** Schedule follow-up contact, face to face or by telephone, preferably within the first week after the quit date.

The “5 R's,” Relevance, Risks, Rewards, Roadblocks, and Repetition, are designed to motivate smokers who are unwilling to quit at this point.

**Relevance**

Encouragement should be given to the patient to indicate why quitting is personally relevant, and should be as specific as possible. Studies show that giving motivation has the greatest impact if it is relevant to a patient's disease status/ risk, family and/or social situation (i.e., having children in the home), health concerns, age, gender, and other important patient characteristics (i.e. prior quitting experience, personal barriers to cessation).^7^

**Risks**

The dentists should ask the patient to identify potential negative consequences of tobacco use. Suggesting and highlighting things that seem most relevant to the patient, the clinician should emphasize that smoking low-tar/low-nicotine cigarettes or use of other sorts of tobacco (e.g., smokeless tobacco, cigars, and pipes) will not eliminate these risks.

**Rewards**

The dentists should ask the patient to identify the potential benefits of stopping tobacco use.

**Examples of rewards:**

Improved health, food will taste better, Improved sense of smell, save money, better self-image, home, car, clothing, the breath will smell better, can stop worrying about quitting, Set a good example for youngsters, have healthier babies and children, eliminate worry about exposing others to smoke, feel better physically, perform better in physical activities, reduced wrinkling/ageing of the skin.^7^

**Roadblocks**

The dentists should ask the patient to spot barriers or impediments to quitting and note elements of treatment (problem-solving, pharmacotherapy) that could address barriers. Typical barriers might include-Withdrawal symptoms, fear of failure, weight gain, lack of support, depression, enjoyment of tobacco.^7^

**Repetition**

The motivational intervention should be repeated every time an unmotivated patient visits the clinic setting. Tobacco users who have failed in previous quit attempts should be told that most people make repeated quit attempts before they are successful.^7^

## WAYS FORWARD

Tobacco use is still a matter of concern in Nepal. The prevalent compromise on Oral health due to Tobacco cannot be neglected. Evaluation of the current strategies, ongoing actions along with the mentioned protocols should be effectively collaborated. The joint venture of active to passive smokers, local bodies, and the government following the global initiative should be encouraged.

## References

[1] The World Bank. (2021). Prevalence of current tobacco use (% of adults) [Internet].

[2] World Health Organization. (2020). Tobacco [Internet]..

[3] Government of Nepal Ministry of Health and Population. (2021). Brief Profile on Tobacco Control in Nepal..

[4] Khanal GN, Khatri RB (2021). Burden, prevention and control of tobacco consumption in Nepal: a narrative review of existing evidence.. Int Health..

[5] Shrestha G, Neupane P, Lamichhane N, Acharya B, Siwakoti B, Subedi K (2020). Cancer Incidence in Nepal: A Three-Year Trend Analysis 2013-2015.. Asian Pacific Journal of Cancer Care..

[6] American Dental Association. (2019). Tobacco Use and Cessation [Internet]..

[7] New York State Smokers Quitline. (2021). Smoking Cessation with Adult Patients: THE 5-R MODEL for tobacco users unwilling to quit [Internet]..

